# Cuticular Wax Composition of Wild and Cultivated Northern Berries

**DOI:** 10.3390/foods9050587

**Published:** 2020-05-05

**Authors:** Linards Klavins, Maris Klavins

**Affiliations:** Laboratory of Natural Products Research, University of Latvia, Jelgava’s Street 1, LV-1004 Riga, Latvia; maris.klavins@lu.lv

**Keywords:** berry, surface, cuticular, wax, vaccinium, chemical composition, lipids, GC-MS

## Abstract

The outer-most layer of plant surface, the cuticle, consists of epi- and intra-cuticular wax. It protects the plant from dehydration, extreme temperatures and UV radiation, as well as attacks from pests such as molds and bacteria. Berry cuticular waxes are studied to understand the metabolism character (factors affecting wax layer composition in different berry species) and increase the microbial resistance and shelf life of berries. The aim of this study was analysis of the surface wax composition of nine species of wild and cultivated berries from Northern Europe. Cuticular wax analysis were done using gas chromatography-mass spectrometry. A total of 59 different compounds were identified belonging to nine groups of compounds, namely, alkanes, phytosterols, alcohols, fatty acids, phenolic acids, ketones, aldehydes, esters and tocopherols. The analyzed blueberries had the highest amount of wax present on their surface (0.9 mg berry^−1^), triterpenoids were the main wax constituent in these berries, with up to 62% wax composition. Berry species and varieties were compared based on their surface wax composition—similarities were found between different blueberry varieties; however, other berries showed differences based on concentration and composition of cuticular wax.

## 1. Introduction

Plant interaction with biotic and abiotic factors is largely dependent on the plant cuticular waxes, which act as an interface between the plant and the environment. The outermost layer of plant organs—the cuticle, which consist of epi- and intra-cuticular waxes (combined—cuticular wax)—protects the plant from abiotic stresses such as dehydration, extreme temperatures (frost, heat) and other factors presented by long-term environmental changes, like increase in the minimum/maximum temperature or disturbances in the precipitation regime in the growth area. Wild plants often suffer from inevitable abiotic stresses, such as insect attacks, fungi, bacteria and parasites [1].

Cuticular wax is a complex mixture; it contains various aliphatic and aromatic compounds. Plant waxes consist of low- to intermediate-polarity compounds, they are hydrophobic, long-chain (chain length from C12 up to C70) chemical compounds [2]. The main compound classes found in the wax are n-alkanes, fatty acids, primary alcohols, aldehydes, secondary alcohols, ketones, phytosterols and esters. Hydroxyl cinnamic acid derivatives and flavonoids are also part of cuticular wax, their presence attributed to the UV protection of plant organs [3]. Considering the composition of plant waxes, hydrophobic solvents are used for extraction, for example, chloroform, hexane or petroleum ether [4].

The composition of plant cuticular waxes has been previously presented for different plant species and plant organs [4]. Previous study on the *Vaccinium* species’ cuticular wax (blueberry, bilberry and bog bilberry) revealed differences among cuticular wax composition based on the compound groups found in these berries. However, more detailed analysis must be done to identify individual components of cuticular wax or biomarkers, which are possibly responsible for bioactivity or resistance against biotic and abiotic stresses [5]. More recently, research on cuticular waxes has been motivated by possibilities to increase the quality of produce—increasing shelf-life and fruit quality and reducing possible microbial infections [6]. For instance, research on the cuticular waxes in grape cultivars has been done to better understand the role in protection against biotic stresses in these berries, which have an important industrial role in the rural parts of the Southern Europe [7]. Triterpenoids, derived from squalene, are a major compound group found on the surfaces of various fruits and berries; they have numerous biological effects and possible pharmacological activities. Functional foods, nutraceuticals and healthcare products containing plant lipids (waxes) are being developed as innovative, consumer-friendly products [8]. The hydrophobic properties of plant cuticular waxes are being investigated for implementation as part of antimicrobial paints, windshield coatings, stain-resistant textiles, cosmetic ingredients and biodegradable plastics [9,10]. Despite the differences among species, the composition of cuticular wax is conserved throughout the plant kingdom [2].

Modernization of agricultural and food production practices has raised questions regarding the possibility of increasing the transportation durability and shelf-life of produce. An important factor in achieving more robust varieties of commercially important fruits and vegetables is the composition of cuticular wax. As consumers demand pesticide- and fungicide-free fruits and vegetables, a safe alternative to using potentially harmful chemicals would be breeding of plants using knowledge on cuticular wax composition, to achieve and preserve the attractive shape, size and aroma, at the same time avoiding spoilage and transportation damage of the produce. Wild and cultivated berries are a seasonal product, in which post-harvest quality and shelf-life largely depends on the damage dealt to the cuticle during berry picking and handling; therefore, for example, blueberries, which are a popular snack, have undergone breeding programs to increase the thickness of the cuticular wax layer [11]. Knowledge of the cuticular wax composition of wild berries like lingonberries, bilberries and cranberries can give insights into the compound groups responsible for increased resistance to biotic and abiotic stresses.

The objective of this study was to identify cuticular wax constituents of commercially important berries in Northern Europe and to classify and establish relationships at the individual or inter-species level, further identifying the compounds and compound classes that are responsible for species specificity, using comprehensive data about cuticular wax composition.

## 2. Materials and Methods

### 2.1. Plant Material

In this study, nine berry species common in Northern Europe were examined for their cuticular wax composition. Examined berries were bog bilberry (*Vaccinium uliginosum* L.), bilberry (*Vaccinium myrtillus* L.), American cranberry (*Vaccinium macrocarpon*), lingonberry (*Vaccinium vitis-idaea* L.), black crowberry (*Empetrum nigrum* L.), gaultheria (*Gaultheria mucronata*), rowanberry (*Sorbus aucuparia* L.), hawthorn (*Crataegus alemanniensis*) and eight varieties of blueberry (*Vaccinium corymbosum* L.), namely, ‘Blue crop’, ‘Blue gold’, ‘Chandler’, ‘Chippewa’, ‘Duke’, ‘North blue’, ‘Patriot’ and ‘Polaris’. The different blueberry varieties and American cranberries were harvested at a commercial blueberry farm Z/S “Strelnieki” located on the outskirts of town Jurmala, Latvia. Bog bilberries, bilberries, black crowberries and lingonberries were harvested from the forests belonging to Kemeru National Park. Rowanberries, hawthorn berries and gaultheria berries were harvested in the vicinity of the town of Saulkrasti, Latvia. To avoid contamination and possible damage to the outer layer of the berries, they were harvested into glass containers using metal forceps, both previously washed with chloroform (≥99%, Sigma Aldrich, Darmstadt, Germany). In total, approximately 700 berries of each species or variety were harvested; all berries were harvested in the summer/autumn of 2018. After this, the harvested berries were placed into a refrigerated sample box and delivered to the laboratory for immediate extraction of cuticular wax.

### 2.2. Extraction of Cuticular Wax

A modified method for extraction of cuticular wax was done [2] using two extraction solvents, chloroform and a mixture of hexane/ethyl acetate (1:1) (≥99%, Sigma-Aldrich, Germany). Each species of berry was extracted three times using each of the solvent. In total, 6 replicates per berry species were prepared. For extraction, three 100 mL beakers were used. A quantity of 50 mL of extraction solvent was poured into each beaker, which were previously cleaned with the same solvent. For each replicate, a hundred berries were picked from the harvested sample and sequentially dipped one by one into the extraction solvent for 30 s in each of the three beakers containing the solvent. Clean metal forceps were used for the berry dipping. After the berry dipping, all of the contents of the three used beakers were filtered and combined into an evaporation flask. Each beaker was further washed twice with extraction solvent and added to the combined extract. Samples were evaporated under reduced pressure using a Rota-Vap evaporator (Büchi, Essen, Germany). Samples were evaporated to approximately 5 mL and transferred to clean glass tubes. The remaining solvent was evaporated in a water bath (40 °C) (Cole Parmer, Vernon Hills, IL, USA) under a gentle stream of nitrogen until dry. The dried berry cuticular wax samples were stored into a freezer (−20 °C) until analysis.

### 2.3. Analysis Using Gas Chromatography-Mass Spectrometry (GC-MS)

GC-MS sample preparation and analysis were prepared according to a previously published methodology [12]. Briefly, the extracted cuticular wax of each replicate was weighed (approximately 20 mg) into three separate GC vials, and the sample was dissolved in 1300 µL pyridine (Sigma-Aldrich). Silylation was done using 200 µL N,O-bis (trimethylsilyl) trifluoroacetamide, BSTFA (Sigma-Aldrich), and samples were heated for 1 h at 60 °C. The total number of replicates ran on the GC system was 18 per analyzed berry species. GC-MS analysis was performed using a GC-2010 plus coupled with a GC-MS QP-2010 Ultra mass detector (Shimadzu, Kyoto, Japan). The column used was a Restek Rxi^®^-5MS (30 m × 0.25 mm × 0.25 μm; Crossbond ^®^ 5% diphenyl + 95% dimethyl polysiloxane (Restek, Bellefonte, PA, USA) with a working temperature range of 40 to 350 °C. He (Helium) was used as carrier gas with a total flow rate of 10.8 mL min^−1^ and a column flow rate of 0.71 mL min^−1^ (AGA, Latvia). The split ratio was 1:10 and injection temperature 290 °C. The temperature program used was: oven temperature 200 °C (2 min) increased to 250 °C at the rate of 30 °C min^−1^ and held for 7 min then increased to 310 °C at the rate of 10 °C min^−1^ and kept for 14 min. Injection of 1.0 μL sample was performed using an autosampler. Mass selective detector with quadrupole mass analyzer was used with electron impact (EI) ionization, with an ionization voltage of 70eV. The ion source temperature was 230 °C and the interface temperature was 290 °C. Identification of the compounds separated in the GC was performed using Shimadzu LabSolutions 4.30 software, coupled with the NIST′17 spectral library (NIST, Gaithersburg, MD, USA), and the compound identity was also confirmed using Kovats Retention Indices [13].

Quantification was done by preparing standard solutions of *β*-sitosterol (≥99.0%) (Extrasynthese, Genay, France), heptadecanoate (≥99.0%), 1-dodecanal (≥98.0%), (±)-*α*-tocopherol (99%), 1-octadecanol (99%), and n-tetracosane (≥99.5%) (Sigma-Aldrich) in the concentration range 1.5–500 µg mL^−1^. Each identified substance was quantified using the standard of the respective chemical family.

### 2.4. Data Analysis

Quantitative data on cuticular wax composition was subjected to two-way analysis of variance (ANOVA) to evaluate the differences between the analyzed berries; post-hoc Tukeys HSD was used to distinguish significantly different groups. Principal components analysis (PCA) on the correlation matrix and hierarchical cluster analysis using Ward’s method with the standardized data was performed to evaluate the relationships among various tested berries. Statistical analysis and data visualization was done using SAS JMP^®^, Version 13 (SAS Institute Inc., Cary, NC, USA).

## 3. Results and Discussion

### 3.1. Wax Amounts

Composition of berry cuticular wax was investigated for nine berry species, both grown commercially and found in the wild forests and bogs of Latvia. In commercially important berries, the thickness of the cuticle and higher wax loads of fruit are crucial, as the post-harvest quality is largely dependent on the cuticular composition in order to avoid dehydration and pathogen attacks [14]. The studied berries were chosen to taxonomically cover different families, different genera, and different varieties of berries (interspecies differences) (Table 1). Substances found as part of the cuticular wax were identified and quantified to evaluate variations of composition and contents among berry species.

**Figure 1:**
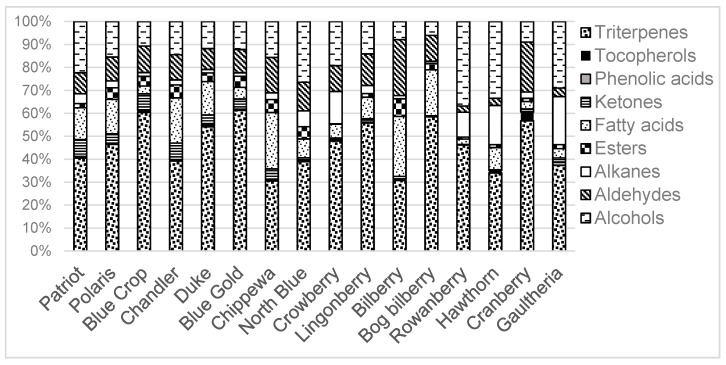
Relative amounts of identified compound classes in studied berries.

The amount of extracted cuticular wax ranged from 0.65 to 0.90 mg berry^−1^ for the investigated blueberry varieties. Lingonberry, American cranberry, black crowberry, hawthorn and rowanberry showed the highest cuticular wax contents, while bilberry showed the least among all of the investigated berries (0.63 mg berry^−1^) (Table 1). These results suggest that the berries that have glossy, smooth cuticular wax layer (not forming wax crystals), like lingonberry, crowberry, rowanberry and cranberries have higher cuticular wax content than the berries that have white, textured cuticular wax layers (crystal forming) [15], like blueberries and bilberries [16]. As proposed by Stevens, Hart and Wollenweber (1995), the glossy mutant of *Sedum rupestre* L. contained 0–5% triterpenoids, whereas the wild type *S. rupestre* with platelet-like wax deposits showed up to 62% triterpenoid content [17]. The glaucous appearance in blueberries could be attributed to the presence of high triterpenoid contents (Figure 1); however, in this study, the morphology of the cuticular wax layer was not investigated. In terms of morphology, the wax layer of blueberries is considered to belong to *β*-diketone tubes (tubule-shaped wax crystals [16,18]), where diketones and triterpenoids were found to be the major compounds [18]. The results reported by Chu and others (2017) are in agreement with our findings—blueberry cultivars have high triterpenoid contents and, among other studied berries, contained high amounts of hentriacontane-10,12-dione (up to 6.0 g 100 g^−1^ extract in ‘North Blue’) [19].

### 3.2. Compound Classes Found in the Cuticular Wax

As part of the berry waxes, 59 different substances (Table A1 and Table A2) were found belonging to nine groups of compounds, namely, alkanes, phytosterols (triterpenoids), alcohols, fatty acids, phenolic acids, ketones, tocopherols and aldehydes (Figure 1). Obtained cuticular wax extracts from different berries show a similar pattern of plant wax constituents, where triterpenoids (up to 62% of total wax content in ‘Blue gold’ and ‘Blue crop’) and alcohols (up to 38% of total wax content in rowanberry) are the major groups of cuticular wax (Figure 1). Aldehydes were found in all of the berries, both, from Ericaceae and Rosaceae families; however, the glossy rowanberry and hawthorn, berries from the Rosaceae family show lower relative aldehyde contents than the rest of the berries, 2–3% and up to 25%, respectively, again suggesting different cuticular wax crystal morphology (Figure 1). As minor groups of compounds found in the berries, phenolic acids and tocopherols were identified; despite the low concentrations of these substances, they have a vital role in the plant–pathogen interaction (Figure 1). Phenolic acids and tocopherols have been reported to have protective abilities against UV radiation and antimicrobial activity, respectively [3,16,20,21,22].

### 3.3. Triterpenoids

**Figure 2:**
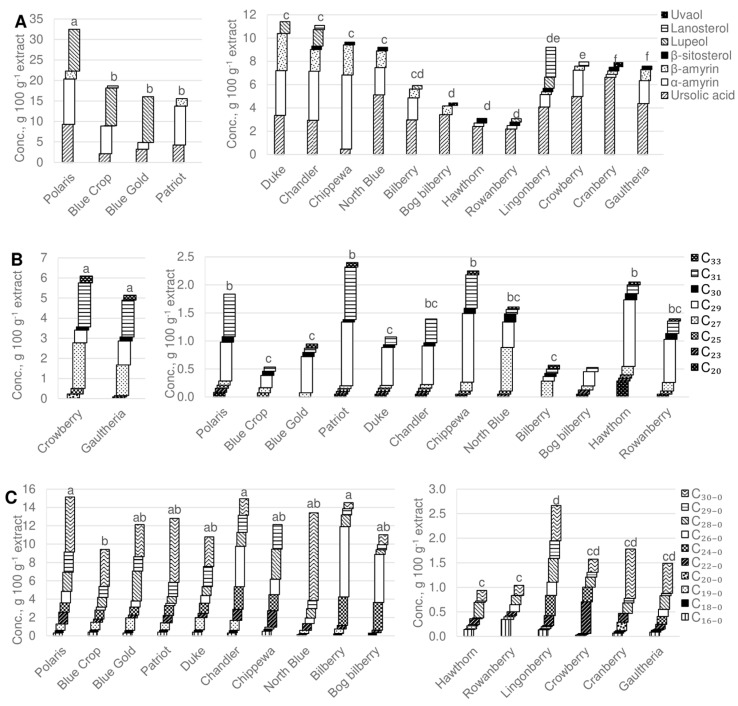
Concentration and composition of sterols (**A**); alkanes (**B**); and fatty acids (**C**) in studied berry waxes. C_16_–C_33_ in (**B**) represent the chain length of alkanes (number of C atoms). C_16-0_–C _30-0_ in (**C**) represent the length of fatty acids, where _−0_ represent the number of double bonds in the fatty acid molecule. Crowberry—black crowberry. Cranberry—American cranberry. Letters above the bars represent significantly different post-hoc pairwise comparison of total concentration of measured substances in respective berry.

Triterpenoids were the most abundant components in the cuticular wax of the studied nine species of berries, varying from 32% (‘Chippewa’) to 62% (‘Blue Gold’) of total wax contents (Figure 1). Eleven different triterpenoids were identified as part of the cuticular wax; the amounts of the seven most abundant triterpenoids are shown in Figure 2.

The triterpenoid acid ursolic acid was found in all of the studied berries in varying amounts. In the blueberry variety ‘Chippewa’ 0.46 g 100 g^−1^ extract ursolic acid was found, while cranberry cuticular wax contained 6.63 g 100 g^−1^ ursolic acid, where it was the predominant triterpenoid (Figure 2A). The analyzed blueberry varieties show different triterpenoid composition patterns. Variety ‘Polaris’ has the highest amount of ursolic acid (9.30 g 100 g^−1^ extract), α-amyrin (11.07 g 100 g^−1^) and lupeol (10.2 g 100 g^−1^) among all of the studied berry waxes (Figure 2A). *α*-amyrin, *β*-amyrin and lupeol are triterpenoid alcohols that are dominant in both the cultivated and wild Ericaceae (Table 1) family berry wax; however, berries belonging to the Rosaceae family, rowanberry and hawthorn cuticular wax contained only ursolic acid, *β*-sitosterol and low amounts of *α*-amyrin. Lanosterol was found in only three berry cuticular waxes—lingonberries (2.58 g 100 g^−1^), ‘Chandler’ (0.34 g 100 g^−1^) and black crowberry (0.35 g 100 g^−1^).

Triterpenoids as part of cuticular wax have been reported previously in a wide variety of plants, for example, apples, cherries, tomatoes, blueberries and plums [9,19,20,21,22,23,24]. Considering the knowledge about the function of cuticular wax, where, for example, alkanes are believed to be responsible for the prevention of water loss, triterpenoids, on the other hand, might play a role in plant–pathogen interaction. Various biological activities and health-promoting effects are attributed to triterpenoid molecules as part of the human diet. As plant defense, triterpenoids have been proven to inhibit germination of the fungus *Alternaria alternata* on Asian pear fruit [25]. As a regulator of plant–pathogen interaction in avocado wax, terpenoids were identified as inducers of appresorium formation [26,27,28]. Triterpenoids were reported to be the main group of compounds in blueberry cuticular wax [19]; it was found that triterpenoids composed 64.2% of total wax, which is in agreement with the reported total wax amounts of specific varieties of blueberries analyzed in this study (up to 62%, Figure 1); the reported dominant triterpenoids are also the same in both studies. During the GC-MS analysis, compounds in minor concentrations with unidentified MS spectra were recorded, possibly belonging to ursane- or oleanane-type triterpenes. The unidentified compounds could contribute to species specificity and cuticular wax protective properties.

### 3.4. Alkanes

Alkanes, which are aliphatic wax constituents with chain length from C_20_ to C_33_, were found in all of the studied berries. Gaultheria, hawthorn, rowanberry and black crowberry cuticular wax were found to contain 21%, 17%, 11% and 14% alkanes of total wax contents, respectively. Blueberry varieties and the rest of the studied berries contained from 1.5% to 7% alkanes of total wax contents (Figure 1). Differences among the relative alkane distributions in berries could be explained by the morphology of the cuticular wax—alkanes are possibly related to the glossiness of the berry, as the glossy berries have higher alkane content than the glaucous berries. The main alkanes found in the cuticular wax of the studied berries were the C_29_ (nonacosane) and C_31_ (hentriacontane) alkanes (Figure 2B). In gaultheria and black crowberry, the C_27_ (heptacosane) alkane was among the dominant alkanes (Figure 2B). The dominant alkanes found on the surfaces of the berries have odd-numbered chain length.

Analyzed blueberry varieties contained from 1.5% to 7% alkanes of total wax, which is higher than that reported by Chu et al., 2017; however, the previously reported alkane composition seems to be similar among the tested varieties, where the odd-numbered alkanes, specifically C_29_ and C_31_, were the dominant alkanes [19]. Alkanes have been found to be part of the outermost layer of many fruits; the dominant C_29_ alkane has been found on the surfaces of plums, apples and cherries [6].

The main function of alkanes as part of the cuticular wax is to control the transpirational water loss of the plant. In a study where two Capsicum species were compared for the post-harvest water loss, it was found that the alkane concentration in the wax was responsible for much lower water loss, rather than the total wax amount [29]. The alkane composition and contents should be interpreted carefully: it has been previously reported that due to increased environmental temperature the concentrations of C_29_ and C_31_ decrease, while those of C_33_ and C_35_ alkanes increase [30]. This implies that the concentration of alkanes is dependent on the environmental stresses provided by temperature fluctuations as the plant adapts to the changing environment. In abnormally hot summers, the alkane ratios can change, therefore giving a false impression of the major alkane concentration in the specific conditions.

### 3.5. Fatty Acids

Saturated fatty acids contribute to 26% of total wax in bilberry and 20% in bog bilberry. Blueberry varieties ‘Chippewa’ and ‘Chandler’ contained 24% and 20% fatty acids of total wax content (Figure 1). Overall, the fatty acid distribution in the studied berries was higher in the Vaccinium species blueberries and bilberries than in the rest of the berries. Additionally, the total amount of fatty acids was higher in blueberries with up to 15.6 g 100 g^−1^ extract in ‘Chandler’. Rowanberry and hawthorn, from the Ericaceae family, contained the least amount of fatty acids with 0.95 and 1.07 g 100 g^−1^ (Figure 2C). As the most abundant fatty acid in the blueberry varieties, triacontanoic acid (C_30-0_) was found; however, this fatty acid was not found in the variety ‘Chippewa’, while the variety ‘North Blue’ contained 9.6 g 100 g^−1^ of this fatty acid. Bilberry and bog bilberry present hexacosanoic acid (C_26-0_) as the major fatty acid, with 7.6 and 5.2 g 100 g^−1^ extract, respectively (Figure 2C).

Identified fatty acids ranged from C_16-0_ to C_30-0_ in different berry species. In blueberry varieties grown in China, the same chain length fatty acids were identified as part of the cuticular wax, with C_30-0_ being the most abundant, followed by C_28-0_ [19]. These long-chain acids have been shown to be responsible for plant–pathogen interaction, either by acting as signaling molecules to initiate defense mechanisms or facilitate fungal infection by allowing the formation of appressoria [20,31]. The variety of fatty acids identified in this study have also been found as part of tomato, Asian pear, apple and orange cuticular wax [6,25,32,33,34,35].

### 3.6. Principal Components Analysis of Wax Profiles

**Figure 3:**
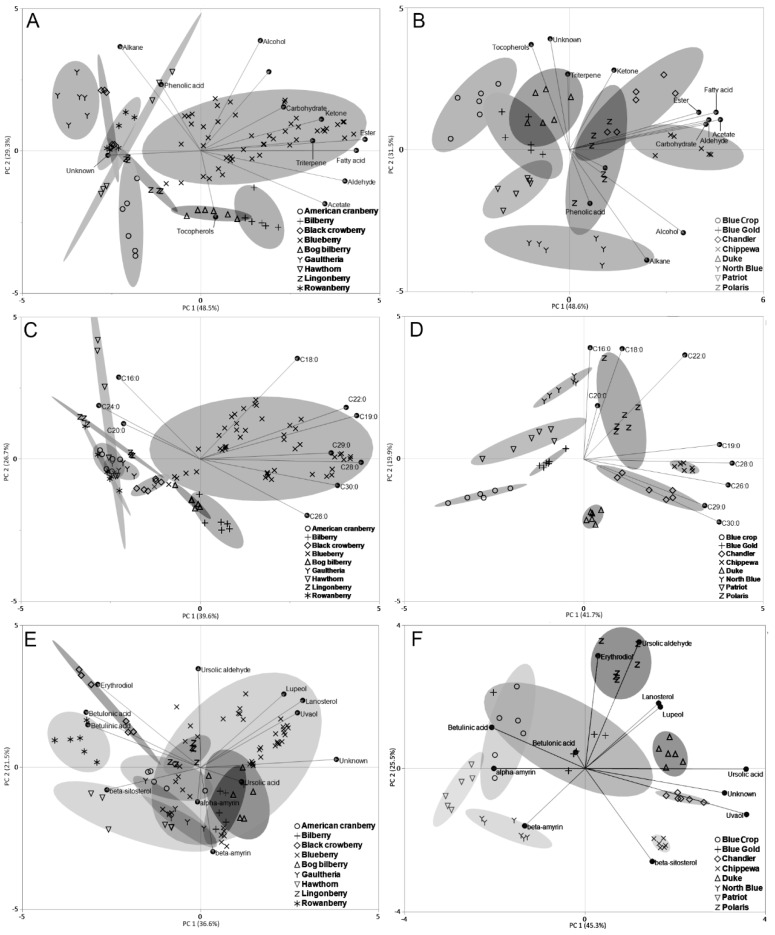
Principal components analysis (PCA) using cuticular wax quantitative analysis of tested berry species and varieties. (**A**) and (**B**) show the PCA scores and loadings plot of different compound groups found in the cuticular wax of tested berry species (**A**) and the different varieties of blueberries (**B**). (**C**) and (**D**) show the PCA scores and loadings plot of the fatty acids found in the cuticular wax extracts of tested berry species (**C**) and the different varieties of blueberries (**D**). (**E**) and (**F**) show the PCA scores and loadings plot of the fatty acids found in the cuticular wax extracts of tested berry species (**E**) and the different varieties of blueberries (**F**). Berries of the same species or variety are grouped by 95% confidence ellipses.

Multivariate analysis, such as Principal Components Analysis (PCA), is often used to visualize complex analytical data and simplify identification of similarities and differences in analyzed clusters or groups of variables. This tool was used to distinguish differences between the analyzed berry wax compositions and to identify compounds responsible for inter- and intra- species variability. In general, maximum variability of data can be explained by analyzing the first two principal components (PC1 and PC2). The first two principal components of the analyzed data explain from 58.1% to 80.1% of the data variability (Figure 3A–F). The PCA plots combined with loadings plots show that the recorded differences can be explained by differences in the concentration of compounds from different groups. Figure 3A shows that such compound groups like fatty acids, triterpenoids and esters are highly related with the clustering of blueberries. On the opposite side of the graph, it can be seen that the clustering of berries like rowanberry, black crowberry and gaultheria is related to the contents of unidentified substances and alkane contents (Figure 3A). Analytical data was recorded for eight different varieties of blueberry—presented data was used to see the inter-species differences among these varieties. When subjected to PCA, it can be seen that the compound groups in the PC1 (like fatty acids, aldehydes and carbohydrates) dominate blueberry varieties ‘Chippewa’, ‘Polaris’ and ‘Chandler’ (Figure 3B). Although the analyzed blueberries come from the same species, it is possible to distinguish these varieties based on the concentrations of various compound groups found in the extracts.

The identified groups of compounds can be used to distinguish the analyzed berry species and varieties from one another to further investigate the specificity of the studied compounds; PCA was done using fatty acid (Figure 3C–D) and triterpenoid (Figure 3E–F) quantification data. The analyzed berry species show separate clusters based on their composition of fatty acids. Blueberry varieties, when pooled in a variety-independent manner, present a large cluster, which indicates dispersion and inter-species variability (Figure 3C). The data on blueberry varieties, when plotted separately, shows that the berries can be split into separate clusters (95% confidence ellipses do not overlap) (Figure 3D). While the clustering of the different species in Figure 3C reveals that rowanberry, gaultheria, cranberries, hawthorn cannot be distinguished based on their fatty acid contents, Figure 3D reveals that each of the analyzed blueberry varieties have specific compositions of fatty acids as part of their cuticular wax. This result implies that the fatty acids and their variation in blueberry cuticular wax can be used as chemometric tools to identify different varieties; however, these results should be interpreted carefully, as the environmental conditions are largely responsible for the composition of cuticular wax and they are subjected to change each harvest season.

The data on various triterpenoids found in berry wax were analyzed using PCA. The main triterpenoids were found in all of the berries in varying concentrations; however, some triterpenoids were found in specific berries, like erythrodiol in black crowberry. Thus, it was postulated that analysis of triterpenoids could indicate species specificity. PCA analysis of all the analyzed berries showed that only rowanberry and black crowberry could be distinguished as a separate cluster in the scores plot (Figure 3E). The same analysis was done on the blueberry varieties, which shows separate clusters of blueberry varieties like ‘Chippewa’, ‘Chandler’, ‘North Blue’ and ‘Polaris’ (Figure 3F). PCA analysis of triterpenoid contents in berries show that, for certain berries, this approach could be used to distinguish between species and varieties of berries. The species-specific triterpenoids, due to low concentrations, have little effect on the final result of the PCA. As mentioned previously, such analysis should be done carefully, as the cuticular wax composition and contents can vary depending on the temperature, moisture and UV radiation that the berries are subjected to throughout the ripening stages.

## 4. Conclusions

Triterpenoids, alcohols and fatty acids were the main compounds identified in the cuticular wax of the nine analyzed berry species. The identified triterpenoids have known biological activity, which implies the possible use of berry wax for use in novel health promoting products. In addition, alkanes and aldehydes, which are attributed with protective ability against environmental or pathogen-induced stress, were identified as part of the cuticular wax. The compounds identified were found to have varying concentrations depending on the berry species. Differences among the berry species were visualized using PCA, which shows that different berry species can be distinguished within the same genus based on analysis of the compound classes found in the wax and that certain classes of compounds can be used to distinguish different varieties of berries within single species. Not much is known about environmental influences on the composition of wax and the wax morphology, therefore, experiments in a controlled environment should be conducted on several berry species in order to understand the influence of a single environmental variable and to pinpoint common pathways of cuticular wax synthesis. The properties of the identified compounds can be attributed to increased resistance and durability of fruits and vegetables; therefore, breeding or genetic engineering can be used to select plants based not only on their size but also on the composition of their cuticular wax, in order to produce berries with natural protection provided by the cuticle.

## Figures and Tables

**Table 1:** Studied berries, their taxonomic relation based on family and genus, and the amount of wax in mg per fresh berry. ± represents the standard deviation of the wax amount (*n =* 6). Means with different letters are significantly different (Tukey’s HSD, *p* < 0.05).

Studied berry	Family	Genus	Variety	Wax, mg Berry^−1^
Hawthorn	Rosaceae	*Crataegus*		1.43 ^ab^ ± 0.09
Rowanberry	Rosaceae	*Sorbus*		1.48 ^ab^ ± 0.09
Gaultheria	Ericaceae	*Gaultheria*		0.65 ^c^ ± 0.02
Black crowberry	Ericaceae	*Empetrum*		1.71 ^a^ ± 0.11
Bog bilberry	Ericaceae	*Vaccinium*		0.95 ^b^ ± 0.09
Bilberry	Ericaceae	*Vaccinium*		0.63 ^c^ ± 0.05
Lingonberry	Ericaceae	*Vaccinium*		1.89 ^a^ ± 0.09
American cranberry	Ericaceae	*Vaccinium*		1.46 ^ab^ ± 0.12
Blueberry	Ericaceae	*Vaccinium*	‘Blue crop’	0.74 ^b^ ± 0.04
			‘Blue gold’	0.67 ^c^ ± 0.03
			‘Chandler’	0.83 ^b^ ± 0.05
			‘Chippewa’	0.90 ^b^ ± 0.07
			‘Duke’	0.57 ^c^ ± 0.02
			‘North blue’	0.65 ^c^ ± 0.02
			‘Patriot’	0.84 ^b^ ± 0.03
			‘Polaris’	0.87 ^b^ ± 0.03

## Appendix A

**Table A1:** Identified compounds found as part of cuticular wax of studied wild berries. ≤LOD—concentration of substance was less than the limit of detection. Concentration of each substance expressed as g 100 g^−1^ of berry cuticular wax extract. Standard error of measurements was determined to be 3–6% (*n* = 18).

Compound Class	Substance	RT, Min	Rowanberry	Hawthorn	A. Cranberry	Gaultheria	B. Crowberry	Lingonberry	Bilberry	Bog Bilberry
**Triterpene**	*β*-sitosterol	21.17	0.291	0.39	0.315	0.3	≤LOD	0.3	≤LOD	≤LOD
	*β*-amyrin	21.38	≤LOD	≤LOD	0.3	0.956	0.356	0.24	0.76	0.747
	*α*-amyrin	21.83	0.3	0.3	0.25	1.958	2.255	1.072	1.89	≤LOD
	Lupeol	22.04	0.3	≤LOD	≤LOD	≤LOD	≤LOD	0.946	0.3	≤LOD
	Lanosterol	22.14	≤LOD	≤LOD	≤LOD	≤LOD	0.35	2.583	≤LOD	≤LOD
	Uvaol	23.83	≤LOD	≤LOD	0.445	≤LOD	≤LOD	≤LOD	≤LOD	0.3
	Erythrodiol	24.43	0.314	0.03	≤LOD	0.004	≤LOD	≤LOD	0.06	≤LOD
	Betulinic acid	25.13	0.232	≤LOD	0.231	≤LOD	≤LOD	1.326	2.811	2.072
	Ursolic acid	25.46	2.188	2.408	6.628	4.379	4.983	4.074	2.968	3.421
	Ursolic aldehyde	26.2	0.589	0.197	≤LOD	0.023	0.832	0.264	0.19	0.418
	Betulonic acid	26.5	≤LOD	≤LOD	0.013	0.184	0.309	≤LOD	≤LOD	1.224
	**Subtotal**		4.214	3.325	8.182	7.804	9.085	10.805	8.979	8.182
**Alkane**	C_20_	4.37	0.05	0.286	≤LOD	≤LOD	0.233	0.05	≤LOD	0.05
	C_23_	7.34	≤LOD	0.05	≤LOD	0.09	≤LOD	≤LOD	≤LOD	0.077
	C_25_	10.03	0.057	0.057	0.057	0.065	0.276	0.057	≤LOD	≤LOD
	C_27_	13.24	0.152	0.151	0.107	1.515	2.268	0.05	0.282	0.07
	C_28_	14.51	0.111	0.012	≤LOD	0.079	≤LOD	≤LOD	≤LOD	≤LOD
	C_29_	15.65	0.771	1.19	0.059	1.196	0.632	0.094	0.084	0.256
	C_30_	16.64	0.105	0.11	0.049	0.203	0.158	0.115	0.057	≤LOD
	C_31_	17.64	0.222	0.158	0.075	1.832	2.188	0.393	0.075	0.075
	C_33_	19.9	0.039	0.05	0.072	0.243	0.342	0.05	0.07	≤LOD
	**Subtotal**		1.507	2.064	0.419	5.223	6.097	0.809	0.568	0.528
**Fatty acid**	Palmitic acid	5.23	0.347	0.143	0.065	0.087	0.025	0.137	≤LOD	≤LOD
	Stearic acid	6.74	≤LOD	0.012	0.032	0.037	0.017	0.022	0.25	0.34
	Nonadecanoic acid	7.81	0.052	0.06	0.02	0.013	0.018	0.038	0.5	≤LOD
	Eicosanoic acid	9.12	0.028	0.025	0.171	0.017	0	0.027	≤LOD	≤LOD
	Behenic acid	12.4	0.077	0.119	0.182	0.092	0.647	0.199	0.397	0.272
	Lignoceric acid	14.93	≤LOD	≤LOD	≤LOD	0.16	0.3	0.414	3.107	3.035
	Hexacosanoic acid	17.03	0.144	0.019	≤LOD	0.148	0	0.27	7.643	5.23
	Octacosanoic acid	18.83	0.186	0.315	0.214	0.277	0.205	0.478	1.286	0.497
	Nonacosanoic acid	20.85	0.002	0.028	0.09	0.051	0.097	0.363	0.68	0.562
	Triacontanoic acid	21.54	0.207	0.218	1.005	0.611	0.263	0.716	0.658	1.076
	**Subtotal**		1.043	0.939	1.779	1.493	1.572	2.664	14.521	11.012
**Acetate**	Hexacosyl acetate	14.64	≤LOD	0.065	≤LOD	≤LOD	0.234	0.414	1.425	4.941
**Alcohol**	Phytol	6.18	≤LOD	0.056	≤LOD	≤LOD	0.033	≤LOD	0.003	≤LOD
	Docosanol	10.93	0.344	0.694	0.007	0.348	0.391	0.221	0.171	1.253
	Tetracosanol	13.88	0.298	0.954	0.037	2.523	0.205	0.024	0.086	0.032
	9-C_27_-ol	15.37	0.309	≤LOD	≤LOD	≤LOD	1.359	1.958	0.164	0.022
	1-Hexacosanol	16.12	0.296	0.593	0.161	0.729	≤LOD	0.028	≤LOD	0.738
	9-C_28_-ol + 10-C_28_-ol	16.37	0.169	0.014	0.041	≤LOD	0.025	≤LOD	≤LOD	0.053
	10-C_29_-ol	17.27	3.704	0.07	0.026	≤LOD	≤LOD	≤LOD	≤LOD	≤LOD
	8,9-C_27_-diol	18.56	≤LOD	≤LOD	≤LOD	≤LOD	≤LOD	≤LOD	0.239	≤LOD
	11-C_31_-ol	19.42	0.188	≤LOD	0.004	0.065	≤LOD	≤LOD	≤LOD	≤LOD
	1-Triacontanol	20.48	0.496	0.042	0.313	0.267	0.094	0.323	0.096	0.75
	1-Dotriacontanol	23.49	0.087	0.011	≤LOD	0.418	≤LOD	≤LOD	0.32	1.528
	**Subtotal**		5.891	2.434	0.589	4.35	2.107	2.554	1.079	4.376
**Aldehyde**	Hexacosanal	12.33	0.045	0.126	0.049	0.058	0.047	0.177	0.048	0.008
	Heptacosanal	13.75	≤LOD	≤LOD	≤LOD	≤LOD	3.908	0.289	0.271	0.063
	Octacosanal	14.4	≤LOD	≤LOD	0.202	0.087	0.235	0.014	0.039	0.033
	Triacontanal	17.09	0.358	0.076	1.056	0.389	0.664	0.432	1.883	5.753
	Dotriacontanal	19.31	0.421	0.175	2.819	0.11	0.599	3.463	2.432	0.296
	**Subtotal**		0.824	0.377	4.126	0.644	5.453	4.375	4.673	6.153
**Carbohydrate**	beta.-D-Allopyranose	4.85	≤LOD	0.559	0.006	0.027	0.027	≤LOD	0.004	0.006
	Myo-Inositol	5.8	≤LOD	0.093	0.019	≤LOD	≤LOD	≤LOD	≤LOD	≤LOD
	Carbohydrate	9.55	≤LOD	≤LOD	0.036	0.01	0.023	0.024	0.099	0.112
	**Subtotal**		0	0.652	0.061	0.037	0.05	0.024	0.103	0.118
**Sesquiterpene**	Abscisic acid	6.61	0.08	0.084	0.144	0.058	0.018	0.011	0.024	0.034
**Ester**	Octacosanoate	19.17	0.186	0.315	0.214	0.277	0.205	0.478	1.286	0.497
**Ketone**	Nonacosane-8,10-dione	18.65	≤LOD	≤LOD	≤LOD	≤LOD	≤LOD	≤LOD	0.287	≤LOD
	Hentriacontane-10,12-dione	21.27	0.014	0.051	0.551	0.615	0.133	0.139	≤LOD	0.493
	**Subtotal**		0.014	0.051	0.551	0.615	0.133	0.139	0.287	0.493
**Phenolic acid**	Syringic acid	4.3	≤LOD	≤LOD	≤LOD	≤LOD	≤LOD	≤LOD	≤LOD	≤LOD
	p-Coumaric acid	4.65	≤LOD	≤LOD	≤LOD	0.173	0.037	0.435	≤LOD	0.021
	Methyl caffeate	5.11	≤LOD	≤LOD	≤LOD	≤LOD	0.053	0.003	0.044	0.023
	Ferulic acid	5.65	0.016	0.013	0.018	0.199	0.009	≤LOD	≤LOD	0.027
	**Subtotal**		0.016	0.013	0.018	0.372	0.099	0.438	0.044	0.071
**Tocopherols**	alpha.-Tocopherol	18.32	≤LOD	0.224	0.622	0.029	0.011	0.153	0.187	0.112
	**Total**		13.775	10.543	16.705	20.902	25.064	22.864	33.176	36.517

**Table A2:** Identified compounds found as part of cuticular wax of studied blueberry varieties. ≤LOD—concentration of substance was less than the limit of detection. Concentration of each substance expressed as g 100 g^−1^ of berry cuticular wax extract. Standard error of measurements was determined to be 3–6% (n = 18).

Compound Class	Substance	RT,min	Patriot	Polaris	Blue Crop	Chandler	Duke	Blue Gold	Chippewa	North Blue
**Triterpene**	*β*-sitosterol	21.17	≤LOD	≤LOD	0.074	0.3	≤LOD	≤LOD	0.235	0.3
	*β*-amyrin	21.38	1.795	1.914	≤LOD	1.865	3.195	≤LOD	2.576	1.439
	*α*-amyrin	21.83	9.519	11.066	6.76	4.211	3.842	1.625	6.374	2.351
	Lupeol	22.04	≤LOD	10.196	9.21	1.418	1.017	11.2	≤LOD	≤LOD
	Lanosterol	22.14	≤LOD	≤LOD	0.508	0.35	≤LOD	≤LOD	≤LOD	≤LOD
	Uvaol	23.83	≤LOD	≤LOD	≤LOD	≤LOD	≤LOD	≤LOD	≤LOD	≤LOD
	Erythrodiol	24.43	≤LOD	≤LOD	≤LOD	≤LOD	≤LOD	≤LOD	≤LOD	≤LOD
	Betulinic acid	25.13	≤LOD	1.994	0.244	5.065	8.33	6.695	1.964	2.165
	Ursolic acid	25.46	4.241	9.304	2.116	2.938	3.356	3.231	0.459	5.115
	Ursolic aldehyde	26.2	≤LOD	≤LOD	≤LOD	0.007	≤LOD	0.012	≤LOD	≤LOD
	Betulonic acid	26.5	≤LOD	≤LOD	≤LOD	≤LOD	≤LOD	≤LOD	≤LOD	≤LOD
	**Subtotal**		15.555	34.474	18.912	16.154	19.74	22.763	11.608	11.37
**Alkane**	C_20_	4.37	0.05	0.082	≤LOD	0.05	0.05	≤LOD	0	0.05
	C_23_	7.34	0.05	0.074	≤LOD	0.05	0.05	≤LOD	0.05	≤LOD
	C_25_	10.03	0.05	0.057	0.074	0.057	0.057	≤LOD	0.057	0.057
	C_27_	13.24	0.05	0.074	0.093	0.064	0.05	0.075	0.778	0.157
	C_28_	14.51	≤LOD	≤LOD	≤LOD	≤LOD	≤LOD	≤LOD	≤LOD	≤LOD
	C_29_	15.65	1.138	0.691	0.219	0.691	0.676	0.648	0.453	1.226
	C_30_	16.64	0.043	0.1	0.075	0.057	0.05	0.075	0.146	0.093
	C_31_	17.64	0.937	0.758	0.075	0.423	0.138	0.075	0.08	0.596
	C_33_	19.9	0.079	≤LOD	≤LOD	≤LOD	≤LOD	0.075	0.044	0.07
	**Subtotal**		2.397	1.836	0.536	1.392	1.071	0.948	1.608	2.249
**Fatty acid**	Palmitic acid	5.23	0.4	0.3	0.4	0.3	0.4	0.3	0.5	0.126
	Stearic acid	6.74	0.25	0.3	0.25	0.3	0.25	0.3	0.25	0.13
	Nonadecanoic acid	7.81	0.769	0.684	0.84	1.084	1.349	1.362	0.195	0.34
	Eicosanoic acid	9.12	≤LOD	≤LOD	≤LOD	≤LOD	≤LOD	≤LOD	≤LOD	≤LOD
	Behenic acid	12.4	0.806	1.304	0.34	1.213	0.457	0.307	1.809	0.74
	Lignoceric acid	14.93	1.076	1.013	0.98	2.466	1.106	0.87	1.738	≤LOD
	Hexacosanoic acid	17.03	0.241	1.266	0.336	4.402	0.833	0.671	1.7	0.607
	Octacosanoic acid	18.83	0.75	2.068	1.062	1.518	0.993	3.26	3.249	1.015
	Nonacosanoic acid	20.85	1.549	2.205	1.2	1.861	2.197	1.56	2.687	0.9
	Triacontanoic acid	21.54	6.958	6.005	4	1.8	3.2	3.5	≤LOD	9.558
	**Subtotal**		12.799	15.145	9.408	14.944	10.785	12.13	12.128	13.416
**Acetate**	Hexacosyl acetate	14.64	0.974	1.013	1.542	1.927	1.294	0.776	1.738	0.549
**Alcohol**	Phytol	6.18	0.162	0.195	0.242	0.531	0.352	0.335	0.291	0.189
	Docosanol	10.93	0.129	0.068	0.122	0.13	0.073	0.128	0.137	0.247
	Tetracosanol	13.88	0.374	0.477	0.094	0.34	0.206	0.167	0.503	0.81
	9-C_27_-ol	15.37	2.407	0.429	0.832	4.373	1.193	1.951	1.414	1.84
	1-Hexacosanol	16.12	≤LOD	≤LOD	≤LOD	0.001	≤LOD	≤LOD	≤LOD	≤LOD
	9-C_28_-ol + 10-C_28_-ol	16.37	≤LOD	≤LOD	≤LOD	≤LOD	≤LOD	0.037	≤LOD	≤LOD
	10-C_29_-ol	17.27	≤LOD	0.259	≤LOD	0.107	0.014	0.24	0.128	0.062
	8,9-C_27_-diol	18.56	≤LOD	≤LOD	≤LOD	≤LOD	≤LOD	≤LOD	≤LOD	≤LOD
	11-C_31_-ol	19.42	0.007	≤LOD	≤LOD	≤LOD	≤LOD	≤LOD	≤LOD	≤LOD
	1-Triacontanol	20.48	1.334	0.869	1.021	1.427	1.237	2.064	2.473	1.52
	1-Dotriacontanol	23.49	≤LOD	≤LOD	0.184	≤LOD	≤LOD	≤LOD	≤LOD	≤LOD
	**Subtotal**		4.413	2.297	2.495	6.909	3.075	4.922	4.946	4.668
**Aldehyde**	Hexacosanal	12.33	0.103	0.17	0.078	0.14	0.015	0.137	0.216	0.119
	Heptacosanal	13.75	0.509	0.605	0.137	0.123	0.269	0.322	0.494	1.234
	Octacosanal	14.4	0.292	0.176	0.132	0.333	0.091	0.165	0.365	0.574
	Triacontanal	17.09	0.566	0.963	0.683	5.581	2.033	0.614	1.7	2.74
	Dotriacontanal	19.31	0.151	2.357	2.219	2.459	2.99	3.074	4.66	1.395
	Subtotal		1.621	4.271	3.249	8.636	5.398	4.312	7.435	6.062
**Carbohydrate**	beta.-D-Allopyranose	4.85	0.011	≤LOD	≤LOD	0.025	0.015	0.014	0.012	0.017
	Myo-Inositol	5.8	≤LOD	≤LOD	0.006	0.021	0.023	0.01	≤LOD	≤LOD
	Carbohydrate	9.55	0.195	0.183	0.121	0.397	0.165	≤LOD	0.13	0.174
	**Subtotal**		0.206	0.183	0.127	0.443	0.203	0.024	0.142	0.191
**Sesquiterpene**	Abscisic acid	6.61	0.048	0.075	0.045	0.056	0.082	0.067	0.037	0.069
**Ester**	Octacosanoate	19.17	0.75	3.068	1.062	1.518	0.993	3.26	5.249	1.015
**Ketone**	Nonacosane-8,10-dione	18.65	≤LOD	≤LOD	≤LOD	≤LOD	≤LOD	≤LOD	≤LOD	≤LOD
	Hentriacontane-10,12-dione	21.27	2.567	2.918	1.334	3.441	2.297	0.583	3.858	6.001
	**Subtotal**		2.567	2.918	1.334	3.441	2.297	0.583	3.858	6.001
**Phenolic acid**	Syringic acid	4.3	≤LOD	≤LOD	≤LOD	≤LOD	≤LOD	≤LOD	≤LOD	≤LOD
	p-Coumaric acid	4.65	0.031	0.004	0.024	0.042	0.034	0.011	0.021	0.02
	Methyl caffeate	5.11	0.055	0.107	0.025	0.086	0.029	0.068	0.083	0.009
	Ferulic acid	5.65	≤LOD	0.002	≤LOD	≤LOD	0.015	≤LOD	≤LOD	≤LOD
	**Subtotal**		0.086	0.113	0.049	0.128	0.078	0.079	0.104	0.029
**Tocopherols**	alpha.-Tocopherol	18.32	0.137	0.218	0.154	0.192	0.234	0.284	0.2	≤LOD
	**Total**		41.553	65.611	38.913	55.74	45.25	50.148	49.053	45.619
